# Repurposing Thioridazine (TDZ) as an anti-inflammatory agent

**DOI:** 10.1038/s41598-018-30763-5

**Published:** 2018-08-20

**Authors:** Mirza S. Baig, Anjali Roy, Uzma Saqib, Sajjan Rajpoot, Mansi Srivastava, Adnan Naim, Dongfang Liu, Rohit Saluja, Syed M. Faisal, Qiuwei Pan, Kati Turkowski, Gajanan N. Darwhekar, Rajkumar Savai

**Affiliations:** 1 Discipline of Biosciences and Biomedical Engineering (BSBE), Indian Institute of Technology Indore (IITI), Simrol, 453552 India; 2 Discipline of Chemistry, Indian Institute of Technology Indore (IITI), Simrol, 453552 India; 3 Centre for Inflammation & Epigenetics, Houston Methodist Research Institute, Houston, Department of Microbiology and Immunology, Weill Cornell Medical College, Cornell University, New York, NY USA; 4 Department of Biochemistry, All India Institute of Medical Sciences (AIIMS), Bhopal, 462020 India; 5 National Institute of Animal Biotechnology (NIAB), Hyderabad, 500032 India; 6 Department of Gastroenterology and Hepatology, Erasmus MC-University Medical Center, 3015 CE Rotterdam, The Netherlands; 7 Max Planck Institute for Heart and Lung Research, Department of Lung Development and Remodeling, 61231 Bad Nauheim, Germany; 8 Acropolis Institute of Pharmaceutical Education and Research (AIPER), Indore, 453771 India; 9 Department of Internal Medicine, Universities of Giessen and Marburg Lung Center (UGMLC), member of the DZL, Justus Liebig University, Giessen, 35392 Germany

## Abstract

Nuclear factor-kB (NF-kB) is a crucial transcription factor in the signal transduction cascade of the inflammatory signaling. Activation of NF-κB depends on the phosphorylation of IκBα by IκB kinase (IKKβ) followed by subsequent ubiquitination and degradation. This leads to the nuclear translocation of the p50- p65 subunits of NF-κB, and further triggers pro-inflammatory cytokine gene expression. Thus, in the need of a more effective therapy for the treatment of inflammatory diseases, specific inhibition of IKKβ represents a rational alternative strategy to the current therapies. A computer-aided drug identification protocol was followed to identify novel IKKβ inhibitors from a database of over 1500 Food and Drug Administration (FDA) drugs. The best scoring compounds were compared with the already known high-potency IKKβ inhibitors for their ability to bind and inhibit IKKβ by evaluating their docking energy. Finally, Thioridazinehydrochloride (TDZ), a potent antipsychotic drug against Schizophrenia was selected and its efficiency in inhibiting IκBα protein degradation and NF-κB activation was experimentally validated. Our study has demonstrated that TDZ blocks IκBα protein degradation and subsequent NF-κB activation to inhibit inflammation. Thus, it is a potential repurposed drug against inflammation.

## Introduction

The nuclear factor-κB (NF-κB) proteins are a family of transcription factors implicated in inflammation, immune response, cell survival and cancer^1–3^. At the basal level, NF-kB is localized in the cytoplasm and its activity is normally suppressed by the interaction with IkB inhibitory proteins, which thereby mask NF-kB nuclear localization signals^4,5^. However, in response to specific external stimuli, including pro-inflammatory cytokines like TNFα, IL1β or endotoxins, viral infection, oxidants, phorbol esters and ultraviolet irradiation, the IkB component of the complex is phosphorylated by IKKβ and degraded, resulting in translocation of NF-kB into the nucleus and the induction of target gene transcription^6–8^. Considering that NF-kB signaling pathways are associated with a large number of inflammatory diseases including arthritis, cancer, and atherosclerosis, hence IKKβ represents a pivotal therapeutic target in the NF-κB pathway^4,9,10^.

Structure-based drug design has enriched the discovery of novel inhibitors in the last few years, for instance, through computational analysis of the novel compounds^11–13^. These include screening both synthetic and natural analogs. In spite of the identification of novel IKKβ inhibitors, none has been developed into clinical treatment^14,15^. Although several synthetic compounds have been shown to be effective in experimental models, however, they did not show much progress in further clinical development^15^. Natural products show less side-effect but low efficacy due to various reasons. For example, resveratrol is a potent anti-inflammatory agent but requires high doses^16,17^. The low absorption profiles of resveratrol pose a challenge for the therapeutic application. To circumvent these issues, we hypothesized the feasibility of repurposing existing drugs as IKKβ inhibitors. We utilized the structure-based drug discovery strategy to screen compounds from already approved FDA drug database employed in ZINC server^18,19^. After initial screening, we compared the docking efficiency of identified candidates with the existing well-known IKKβ inhibitors. Finally, we short-listed Thioridazine (TDZ) as the most potent IKKβ inhibitor. Importantly, we have experimentally demonstrated the inhibition of IKKβ phosphorylation and TNFα-induced NF-κB signaling *in vitro*, and the anti-inflammatory efficacy in mice by TDZ treatment.

## Materials and Methods

### Docking-based virtual screening

The crystal structure of IKKβ in complex with a synthetic inhibitor (PDB ID: 3RZF)^20^ was used as the model for the structure-based screening. DockBlaster^21^ was used for the prediction of novel inhibitors utilizing virtual screening and docking calculations. Dock Blaster is an online server that selects and scores thousands of compounds deposited in the ZINC database^18^ based on their binding energy with the protein target. Its flexible-ligand sampling algorithm superimposes atoms of the docked molecule as per the given binding site, which represents positions for binding ligand atoms. For the input structure, we specified 3RZF as the structure for the receptor protein while the binding site was specified using the bound inhibitor grid coordinates for the docking calculation. As we were interested in retrieving hits solely for drug repurposing, we selected the ZINC subset [http://www.epa.gov/nheerl/dsstox/] containing FDA approved entries. In DockBlaster, the ligand selection was performed on the basis of bin size, distance tolerance up to 2.0 Å, areceptor-ligand configuration that passes electrostatic, Van der Waal interactions complementarity and corrected for ligand desolvation. The high-scoring ligand conformation was minimized with 100 steps of simple rigid-body minimization.

After the completion of Dock Blaster virtual screening, we retained the top 5 compound hits as sorted by their Dock Blaster energy-rank (Supplementary Table 1). Next, we employed AutodockVina interfaced with mcule program^22^ for the docking calculations of these 5 compounds along with the docking of 8 well-known high potencies IKKβ inhibitors (Supplementary Table 2). We employed mcule program for this docking run in spite of the initial screening with Dock Blaster because the latter does not take user-specified input files. Similar starting structure was used for the dockings as discussed for the Dock Blaster above. The mcule docking program does not identify active site using the co-crystallized ligand, hence, we manually specified the binding site by providing the grid center coordinates of the bound ligand; 90.978742, −23.192645, 54.212806. The docking calculations resulted in the retrieval of docked poses of all the 13 compounds which were further compared for evaluation of binding efficacy. After evaluating the dockingscore of all compounds, two highest scoring compounds from the FDA approved database; 1530695 (Thioridazine Hydrochloride) and 3830847 (Flubendazole), were further evaluated and compared with one of most potent compound of IKKβ- derivative of Bayer Compound A with a Ki of 2 nM and a VinaDock Score of −8.4 for IKKβ binding using the Swiss dock server^23^. For final docking and validation, we used SwissDockprogrambased on EADock dihedral space sampling (DSS) or EADock DSS. This program is built on the most efficient features of EADock2, a user-friendly program that utilizes algorithmically flexible and accurate approaches based on a hybrid sampling engine and a multiobjective scoring function. EADock DSS generates multiple binding modes as generated either in a box (local docking) or in the vicinity of all target cavities (blind docking). Furthermore, it calculates the CHARMM energies and clusters the binding modes of most favorable energies using FACTS software. Similar starting structure has been used for SwissDock as used for the above two dockings. However, being a blind-docking run, binding site was not specified in this case. Finally, SwissDock grouped the most favorable clusters for further interpretation. UCSF-Chimera^24^, a molecular visualization software as well as Discovery Studio 4.3 (BIOVIA Discovery Studio 2017 R2, 2016) was used to visualize and analyze all structure-based features.

### Murine model

Briefly, the wild-type Swiss albino mice were obtained from Animal Facility Division, Defence Research and Development Establishment (DRDE, Gwalior, India). The mice were all male of 6–8 weeks old, each weighing ~30 g. The animal study was approved by the Institutional Animal Ethics Committee (IAEC) of Acropolis Institute of Pharmaceutical Education and Research and conducted in accordance with the policies of Committee for the Purpose of Control and Supervision of Experiments on Animals (CPCSEA), Govt. of India. Mice were kept under the standard conditions of temperature (20–25 °C) and relative humidity (55–60%) with a 14:10 h of light and dark cycle and had ad libitum access to purified water and diet of dry pellets.

To study the therapeutic efficacy of Thioridazine in LPS-injected mice, the mice were first acclimatized and then randomly distributed in equal groups as described below. In total, four groups were created (2 control and 2 experimental) with 14 mice in each assigned group. Briefly, Thioridazine (Cayman chemicals, USA) was dissolved in a solution of dimethyl sulfoxide (DMSO) (MP Biomedicals) and Milli-Q Water (in the ratio of 1:9) and all the 14 mice in thioridazine group were administered with 0.5 ml of 500 µg/mice dose of thioridazine by intraperitoneal injection. The control groups received 0.5 ml of 1X PBS and/or DMSO and Milli-Q water solution (ratio 1:9). After 1 hr, the mice were injected with Escherichia coli lipopolysaccharide (*Escherichia coli*, 0111:B4; Sigma-Aldrich). In agreement with previously reported dose for lung injury/septic inflammation model^25^, all the animals in experimental groups were induced by intraperitoneal injection of 30 mg/kg of LPS diluted in 0.5 ml of 1X PBS for each mouse. The protective effect of thioridazine was determined by recording the mortality of mice up to 96 hrs. After 96 hrs, survived mice who were moribund were euthanized by giving anesthesia followed by cervical dislocation. Both left and right lungs were removed and fixed for Hematoxylin and eosin (H&E) staining or immunohistochemistry with antibodies.

### Cell culture

Mouse RAW 264.7 macrophages were obtained from National Centre for Cell Science (NCCS), Pune, India. Macrophages were cultured in Dulbecco’s minimal essential medium (DMEM) (Invitrogen) supplemented with 10% heat-inactivated FBS (Life Technologies) along with 100 U/ml penicillin and 100 µg/ml streptomycin (Invitrogen). Cells were cultured in a humidified incubator with 5% CO_2_ at 37 °C. Macrophages were seeded in multi-well plates and incubated overnight prior to treatment with LPS (250 ng) and Thioridazine (10 µM) for indicated time points.

### Immunoblotting

For immunoblot analysis, cells were lysed in RIPA buffer (Thermo Scientific) containing protease and phosphatase inhibitors (Sigma-Aldrich), resolved on NuPAGE 4–12% gels (Novex, Life Technologies), and blotted and probed with the following antibodies against IκB-α, β-actin (Santa Cruz Biotechnology, Inc.), p65 (Cell Signaling Technology). After secondary HRP staining (Santa Cruz Biotechnology, Inc.), chemiluminescence was detected using HRP Substrate (Serva). Images were digitally scanned (Image Quant LAS 4000) and analyzed using ImageJ (National Institutes of Health; Schneider *et al*., 2012).

### Immunofluorescence staining

RAW 264.7 macrophages (1 × 10^4^) were seeded on sterile coverslips. Prior to treatment, cells were allowed to synchronize by growing them in Dulbecco’s minimal essential medium (DMEM) serum-free media overnight. Cells were treated with Thioridazine (10 µM), 30 minutes prior to lipopolysaccharide (LPS) treatment (250 ng/mL) for 10 and 30 minutes respectively. After treatment, the media was aspirated and washed three times with 1X phosphate buffered saline (PBS). The cells were fixed with 4% paraformaldehyde and again washed three times with PBS (1x). Further cells were permeabilized with 0.1% Triton-X-100 for 10 minutes at room temperature. Cells were then blocked with 5% BSA in 1X PBS for 1 h at room temperature followed by incubation with primary antibody for IkB-α (sc-371, Santa Cruz Biotechnology, Inc.) for 1 h (1/200 in TBST buffer). After washing with 1X PBS, cells were stained with secondary antibody Chicken-anti rabbit-Alexa Fluor 594 (Molecular Probes-A21442) for 1 h. Nuclear counterstaining was done using DAPI (Thermo Fisher Scientific) according to manufacturer’s instructions. Stained cells were analyzed by Olympus confocal laser scanning microscope at 40X magnification and 6 times zoom.

### Immunohistochemistry

Lung tissues isolated from surgically resected specimens were fixed in 40% paraformaldehyde for 1 h and then embedded in paraffin. 5 μm-thick sections were deparaffinizedin xylene and rehydrated through a gradient series of ethanol. After that, it was rinsed in distilled water and placed in 1X PBS. Further, heat-induced antigen retrieval was carried out by a 10-minute incubation at 90 °C in Tris (10 mM)-EDTA (1 mM) buffer (pH 9.0) and subsequently all sections were blocked with 5% BSA (MP Biomedicals, USA). Next, tissue sections were incubated with primary monoclonal rabbit anti-mouse IkB-α antibody (sc-371, Santa Cruz Biotechnology, Inc.) for1 h at room temperature followed by fluorescently tagged chicken anti-mouse IgG secondary antibody incubation (Santa Cruz Biotechnology, Inc.) for 1 hr. Nuclear counterstaining was done using DAPI (Thermo Fisher Scientific) according to manufacturer’s instructions. Stained tissue was analyzed by Olympus confocal laser scanning microscope at 20X magnification and 3.5 times zoom.

### RNA isolation and RT-PCR

Total RNA was isolated from cultured RAW 264.7 macrophages and snap frozen lung tissue by RNAiso Plus reagent (Takara Bio Inc.) according to manufacturer’s instructions. The concentration and purity of extracted-RNA were determined by the ratio of absorbance readings at 260 nm and 280 nm (A260/A280). Total RNA (1 μg) was reverse transcribed (RT) using the iScript™ cDNA Synthesis Kit (Bio-Rad) according to the manufacturer’s specifications. Real-time PCR was performed using SYBR® Select Master Mix (Applied Biosystems) in StepOnePlus Real-Time PCR Systems (AppliedBiosystems). Briefly, the reaction conditions consisted of 0.5 μl of cDNA and 0.2 μM primers in a final volume of 20 μl of supermix. Each cycle consisted of denaturation at 95 °C for 15 s, annealing at 58.5 °C for 5 s and extension at 72 °C for 10 s, respectively. The experiment was performed by three independent experiments in triplicate. The following gene-specific primer sets (each 10 picomoles) were used to amplify the target genes: IL-1β forward 5′-TGCCACCTTTTGACAGTGATG-3′; IL1β reverse 5′-AAGGTCCACGGGAAAGACAC-3′; IL6 forward 5′-GCCTTCTTGGGACTGATGCT-3′; IL6 reverse 5′-TGCCATTGCACAACTCTTTTC-3′; NOS2 forward 5′- GGCAGCCTGTGAGACCTTTG-3′; NOS2 reverse 5′- GCATTGGAAGTGAAGCGTTTC-3′; TNFα forward 5′-AGGCACTCCCCCAAAAGATG-3′; TNFα reverse 5′-CCACTTGGTGGTTTGTGAGTG-3′; GAPDH forward 5′-GGTCCTCAGTGTAGCCCAAG-3′ and GAPDH reverse 5′-AATGTGTCCGTCGTGGATCT-3′.

### ADP-Glo IKKβ-kinase assay

White low-volume 384-well polystyrene plates (OptiPlate-384, PerkinElmer, Waltham, MA) were used for the ADP-Glo assay. IKKß Kinase assay was performed according to the manufacturer protocol (Promega #TM313). ATP/ADP Standard curve was performed with different concentrations of ATP/ADP. The assay was performed in two steps; first, after kinase reaction, using 20 ng IKKβ, 0,2 µg IKKtide, 25 µM ATP and different concentrations of Inhibitor (1 nM, 10 nM, 100 nM,1 µM,10 µM) an equal volume of ADP-Glo™ Reagent was added to terminate the kinase reaction and deplete the remaining ATP. In the second step, the Kinase Detection Reagent was added, which simultaneously converts ADP to ATP, which can be measured using a coupled luciferase/luciferin reaction. The luminescence was quantified using a Multimode Microplate Reader (Tecan M200 Pro).

## Results

### Drug repurposing by virtual screening

**Figure 1:**
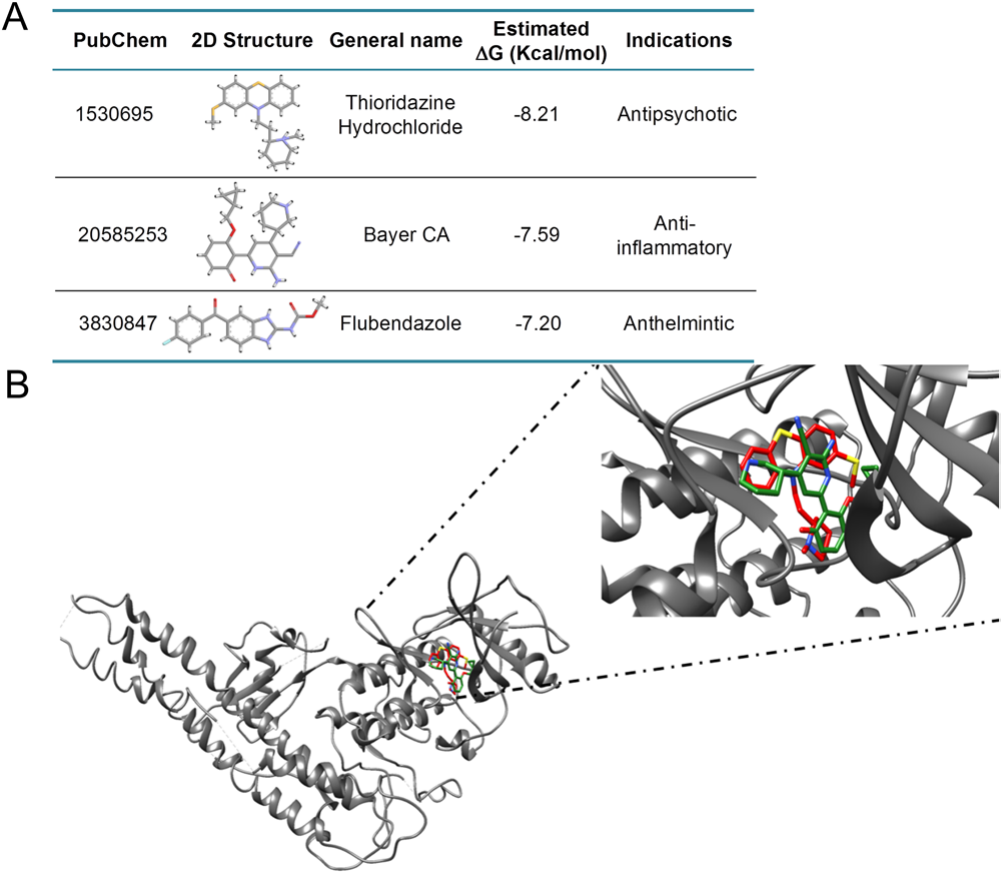
Docked conformations of Bayer CA and 1530695 in the IKKβ binding site. The grey ribbon is the IKKβ structure, while the red and green sticks are Thioridazine (TDZ: 1530695) and Bayer CA (20585253) respectively. The right side figure is the zoomed image of the IKKβ binding site bound with the inhibitors. The inserted table indicates the ∆G values of ZINC FDA compound TDZ and Bayer CA using the Swissdock server.

In this strategy, we used the crystal structure of IKKβ bound with a synthetic inhibitor for the structure-based screening protocol utilizing the Dock Blaster docking program. The crystal bound inhibitor itself was specified for the binding site identification. Drug repurposing has been done using the ZINC enabled FDA database implemented in Dock Blaster. The top 5 hits as ranked by Dock Blaster scoring function are tabulated in Supplementary Table 1. They were further docked using the Autodockvina program and compared with already known high-potency IKKβ compounds (Supplementary Table 2). These include Bayer Compound A, ML-120B, SC-514, TPCA-1, IMD-0354, BMS-345541, Wedelolactone, and GSK-‘azaindole-7. To compare binding energy, we docked all 13 compounds using the AutoDockVina software through mcule 1-click docking server. Mcule program provides a  highest negative score as the highest ranking pose. Mcule ranked ZINC compounds 1530695 as well as 3830847 as the top binders with a Dock Score of 8.6 and 8.8 respectively, which are better than the score of the most potent IKKβ inhibitor-derivative of Bayer Compound A (Ki of 2 nM) with a dockscore of −8.4. Further, we recalculated the binding affinity of these three compounds; including 1530695, 3830847 and Bayer Compound A as a final screen to identify the strongest binder for IKKβ, using the SwissDock server. 1530695 (Thioridazine, TDZ) outperformed both 3830847 and Bayer CA in estimated ΔG value for IKKβ (Fig. 1). Hence, we finally selected 1530695 for further investigation. The comparative docked conformations of both 1530695 and Bayer CA in the IKKβ binding site are depicted in Fig. 1. Both compounds exquisitely superimposed with each other in the binding space of IKKβ, further validating the efficiency of the docking program.

### Thioridazine (TDZ) treatment protected mice from septic injury and mortality

**Figure 2:**
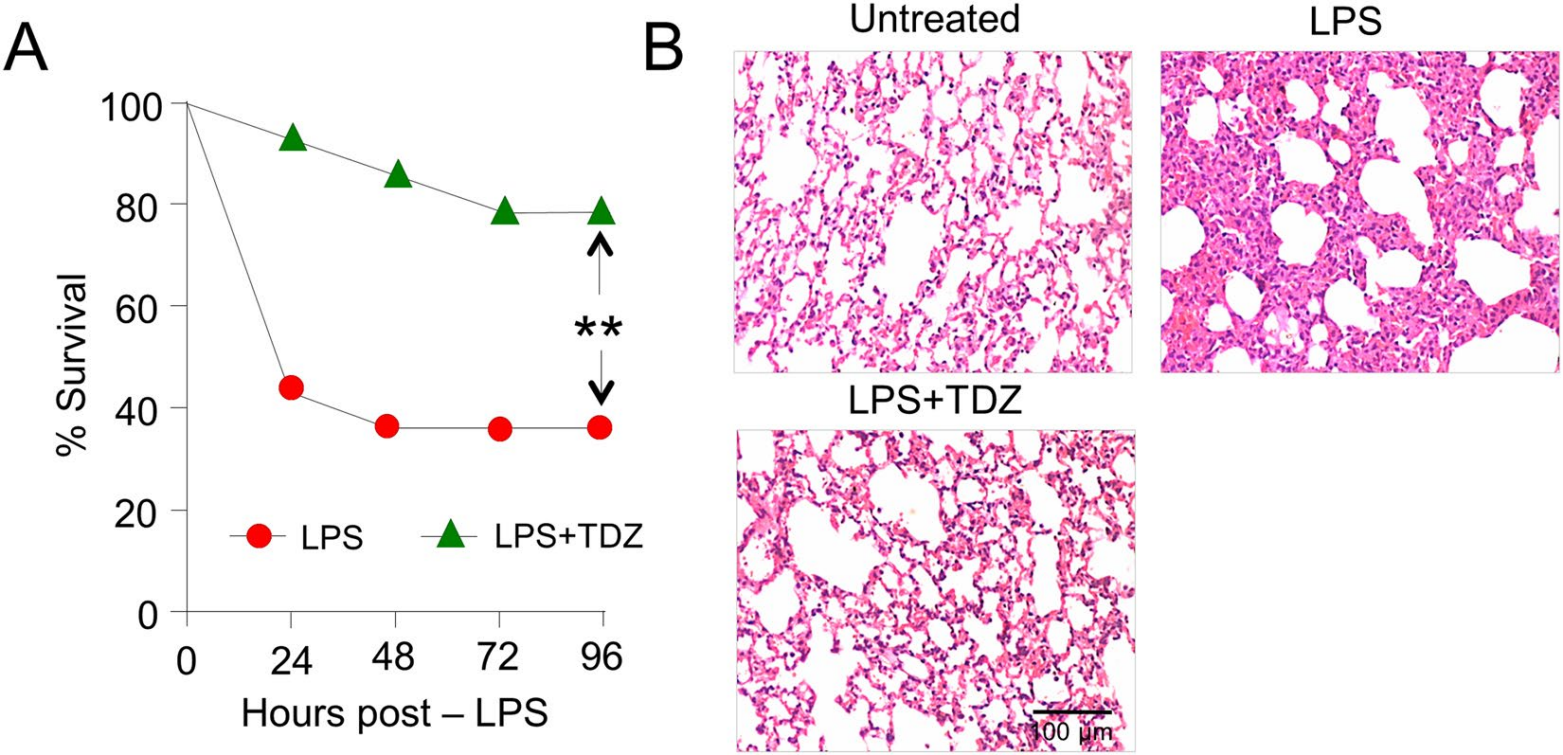
TDZ protects against model septic injury. **(A)** Survival curve of mice (n = 14, each group) treated with LPS alone (20 mg/kg, i.p.) or in combination with TDZ (15 mg/kg, i.p.) 1 h prior to LPS. **(B)** Lung histological analyses were performed using hematoxylin and eosin (H&E) staining in control and TDZ pre-treated mice 8 h after LPS challenge. Representative images from three mice per phenotype analyzed in three independent experiments are shown. Scale bar = 100 µm. Significance was determined using Fisher’s exact test. ^**^P < 0.001.

We employeda septic inflammation mouse model induced by IP injection of LPS lethal dose (25 mg/kg) As expected, all the mice in control groups (n = 14 per group) that received PBS and/or DMSO + Water were survived. Mice that received only LPS (n = 14) showed that 57% (n = 9) of them succumbed within 24 hrs; 36% mice were survived over a period of 96 hrs. In contrast, the mice that were treated with TDZ (n = 14) had a survival rate of 93% (n = 13) and 86% (n = 12) within 24 hrs and 48 hrs, respectively. In total, 79% survival rate was achieved over the lethal dose of LPS at the end of 96 hrs (Fig. 2A). The survival rate of TDZ group mice confers the high efficacy of administered dose (500 µg/mice) of TDZ and suggest it as a potent anti-inflammatory target specific drug against the septic inflammation model.

**Figure 3:**
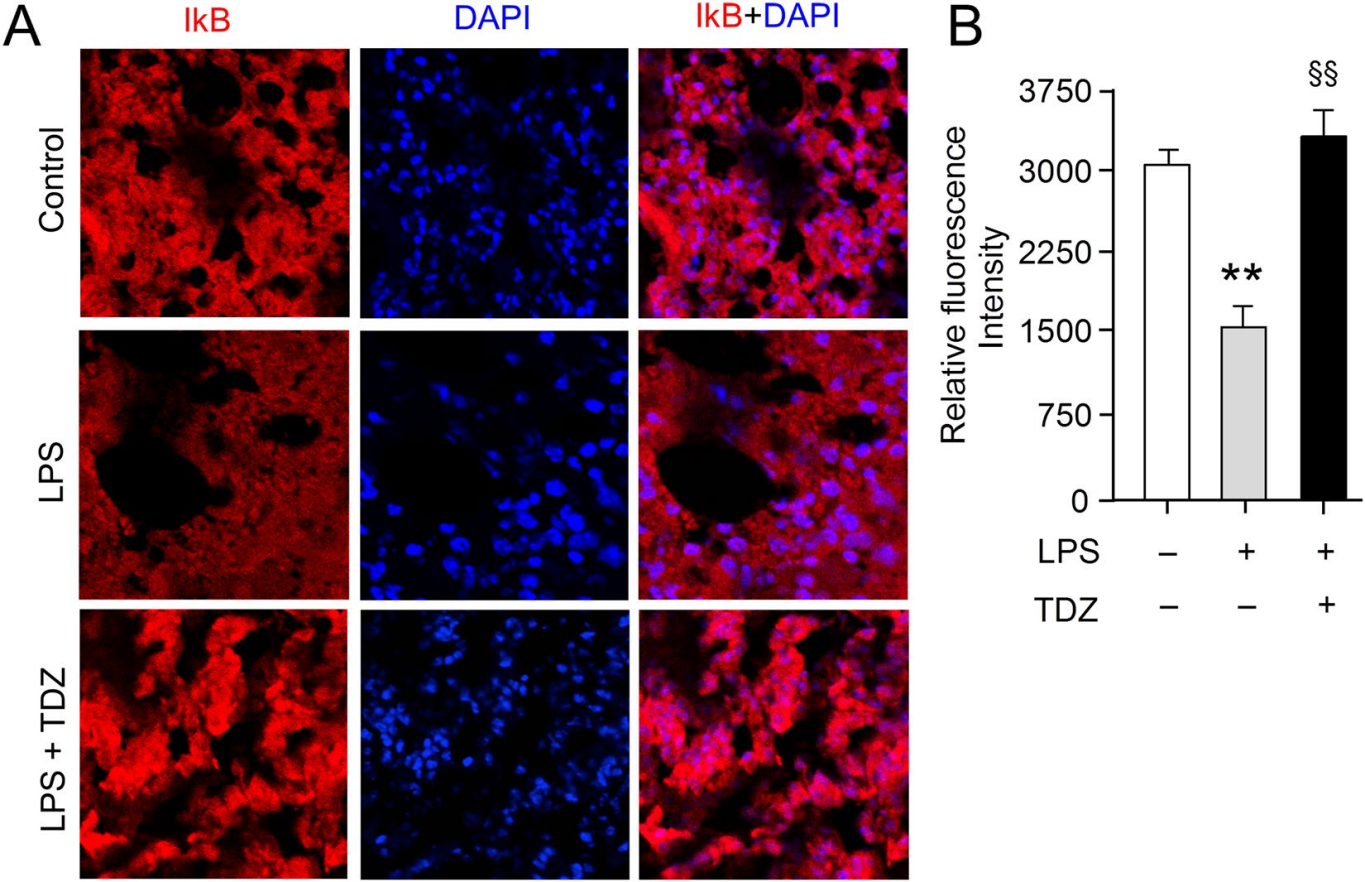
TDZ inhibits LPS-induced IkB degradation in mouse lung tissue. **(A)** Immunofluorescent staining was performed in lung tissue sections of mice treated with LPS alone (20 mg/kg, i.p.) or in combination with TDZ (15 mg/kg, i.p.) 1 h prior to LPS. Lung tissue sections were immunostained with anti-IkBα antibody and detected by the fluorescently tagged secondary antibody. Representative images from three mice per phenotype analyzed in three independent experiments are shown. Scale bar = 100 µm. (**B**) Quantitation of IkB staining. Total 10 slices per treatment condition were used to take the average intensity of IkB. Significance was determined using Fisher’s exact test. ^**^P < 0.005; ^§§^P < 0.001.

The lung tissues were examined under the light microscope (magnification, 20×). The morphology of pulmonary tissue was normal with alveolar septa, pulmonary capillaries, epithelium lining the airways preserved in the control group (DMSO + water) of mice. Lung tissues of LPS-injected mice were distorted by infiltration of lymphocytes and neutrophils, pulmonary tissue edema, alveolar epithelium cells breakage, mucus-filling airways (serous effusion), thickening of the alveolar septum, and interstitial scarring. In contrast, the pre-treatment of TDZ in LPS-injected mice have shown recovery in lung tissue with features similar to the control group. The epithelium lining of airways was intact, showing normal alveolar septum and no serious effusion (Fig. 2B). The immunofluorescent staining using anti-IκBα antibody on mouse lung sections revealed that TDZ treatment of mice inhibited IκBα degradation (Fig. 3).

### Decreased IκB degradation is directly correlated with p65 nuclear translocation following treatment with TDZ

**Figure 4:**
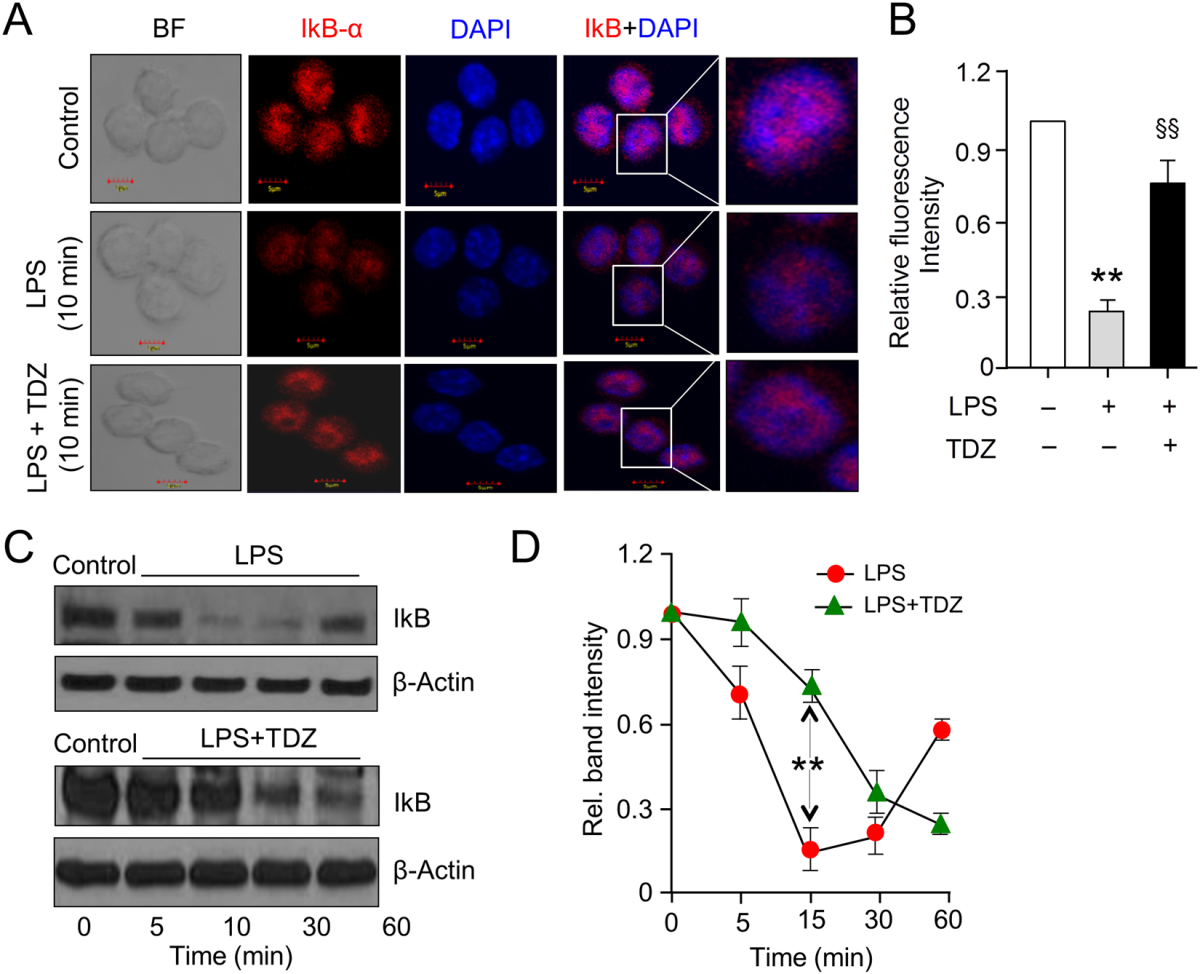
TDZ inhibits LPS-induced IkB degradation in RAW 264.7 macrophages. **(A)** Immunofluorescence confocal microscopy of IkBα in RAW 264.7 macrophages. RAW 264.7 macrophages were treated with an anti-IkBα antibody to determine the level of IkB (red). Nuclei were counterstained with DAPI (blue) and slides were visualized using confocal microscopy. Merged images of the red and blue fluorescence are shown. Original images ×800 for all panels. The images are representative of three independent preparations. **(B)** Bar graph showing the mean fluorescent intensities of IkBα quantified from the images shown in A. **(C,D)** RAW 264.7 macrophages were treated with LPS (250 ng/ml) with or without TDZ (10 μM) for indicated time points and RIPA lysed cells were subjected to western blotting using antibodies specific for IkBα and β-actin. All data are representative of three independent experiments; all are presented as mean ± SD. P Significance was determined using Fisher’s exact test. ^**^P < 0.005; ^§§^P < 0.001.

The activation of IKK phosphorylates the inhibitory IκBα protein, leading to its degradation and the subsequent release of the NF-κB complex, which then translocates into the nucleus to activate the transcription factors of the pro-inflammatory genes. To examine the IκBα degradation, we performed immunocytochemistry on RAW 264.7 macrophages treated with LPS alone or in combination with TDZ for indicated time points. The fluorescent images show that for untreated macrophages, IκBα was sequestered in the cytoplasm; whereas in LPS-stimulated macrophages IκBα was degraded and treatment with TDZ restored the cytoplasmic expression of IκBα (Fig. 4A,B). Immunoblot analysis revealed that IκBα degradation starts 5 min after LPS treatment up to 15 min. However, IκBα degradation is gradually restored 30 min onwards with complete restoration up to 120 min. Contrary to the LPS effect, in presence of Thioridazine along with LPS, IκBα degradation is delayed up to 30 min. Degradation begins to post 30 min followed by gradual restoration 90 min onwards (Fig. 4C). Densitometric analysis of immunoblot represents a similar pattern of IκBα degradation and restoration, showing the effect of Thioridazine on IκBα expression (Fig. 4D). The delayed degradation of IκBα accounts for the effect of TDZ, which is due to IKK inhibition. IκB degradation and free NF-κB subunits translocation to the nucleus have been widely studied in inflammatory and innate immune response to a stimulus. Stimulation of RAW 264.7 macrophages with LPS at 10 minutes, shows activation of inflammatory pathway comprising phosphorylation-induced, proteasome-mediated degradation of IκB.

### TDZ inhibits the expression of pro-inflammatory cytokines

**Figure 5:**
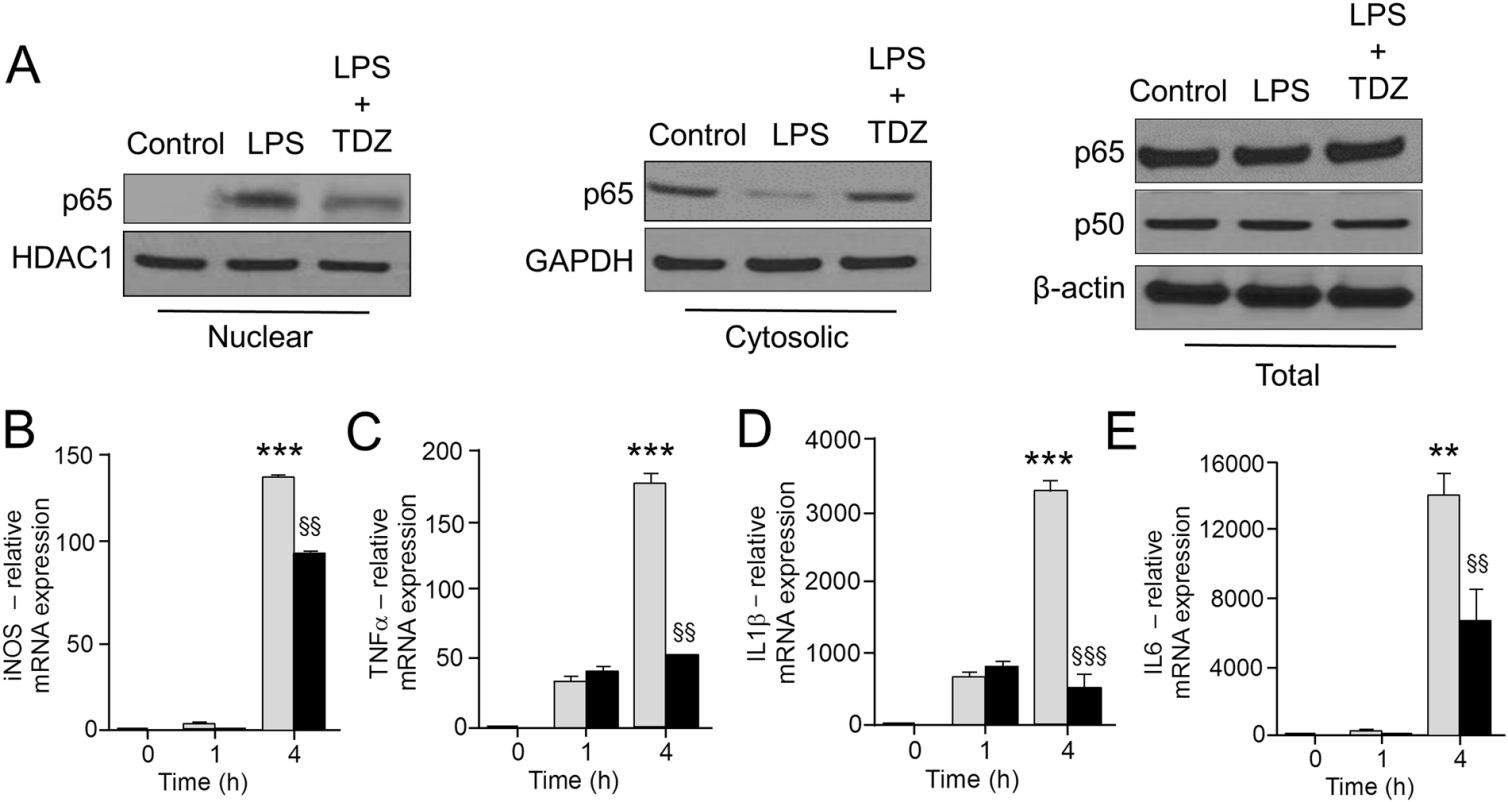
TDZ inhibits NF-κB nuclear translocation and suppressed the induction of NF-kB dependent pro-inflammatory signatures in RAW 264.7 macrophages. **(A)** Isolated cytoplasmic and nuclear fractions were probed for p65 by immunoblot before and after LPS (250 ng/ml) for 15 min, GAPDH and HDAC1 are loading controls for cytoplasmic or nuclear protein, respectively. **(B–E)** qPCR quantification of NF-kB dependent genes including iNOS, TNFα, IL1β, and IL6 treated with LPS or LPS + TDZ for the indicated time. The treatment of macrophages with TDZ significantly suppressed the transcription of NF-kB dependent genes. Values were normalized to GAPDH. P values were determined by Student’s T-test; ^***^P < 0.005, <0.005^**^P < 0.05, ^§§^<0.001.

**Figure 6:**
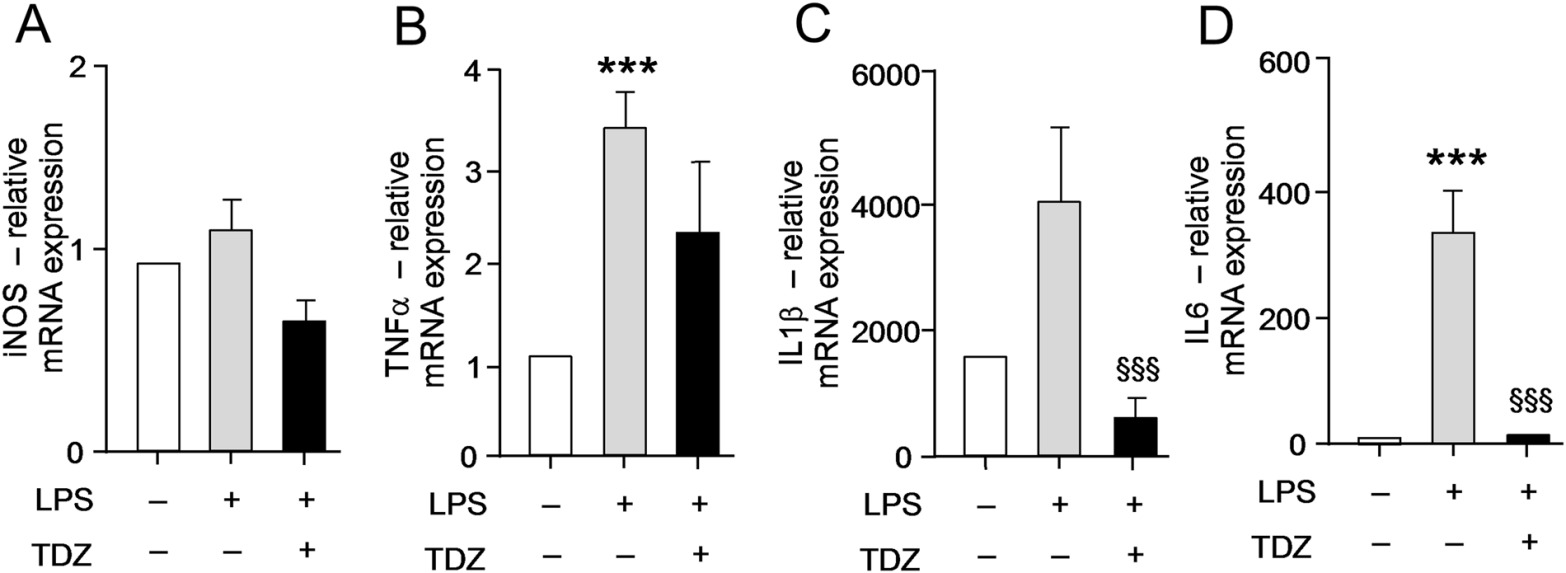
TDZ treatment decreases NF-kB mediated transcriptional activation of pro-inflammatory cytokine expression in the lung tissue. (**A**–**D**) qPCR quantification of NF-kB dependent molecular signatures NOS2 **(A)**, TNFα **(B)**, IL1β **(C)**, and IL6 **(D)** in mice lung treated with LPS or LPS + TDZ for 24 h. TDZ treatment significantly decreased the induction of NF-kB dependent pro-inflammatory molecular markers. Values are mean ± standard error and treatment groups were normalized to GAPDH. ^***^P < 0.05, ^§§^<0.005.

To investigate the inhibitory effect of TDZ on the expression of pro-inflammatory cytokines, RAW 264.2 macrophages were treated with LPS in a time-dependent manner. LPS stimulation shows a significantly higher expression of all the pro-inflammatory markers (TNFα, IL1β & IL6) at different time points (1 h and 4 h). TDZ treatment prior to LPS induction shows a significant decrease in cytokine expression as shown in Fig. 5. We have also confirmed the decreased level of pro-inflammatory cytokines in lung tissues of TDZ treated mice after LPS injection (Fig. 6).

### TDZ inhibits IKK activity by decreasing its phosphorylation

**Figure 7:**
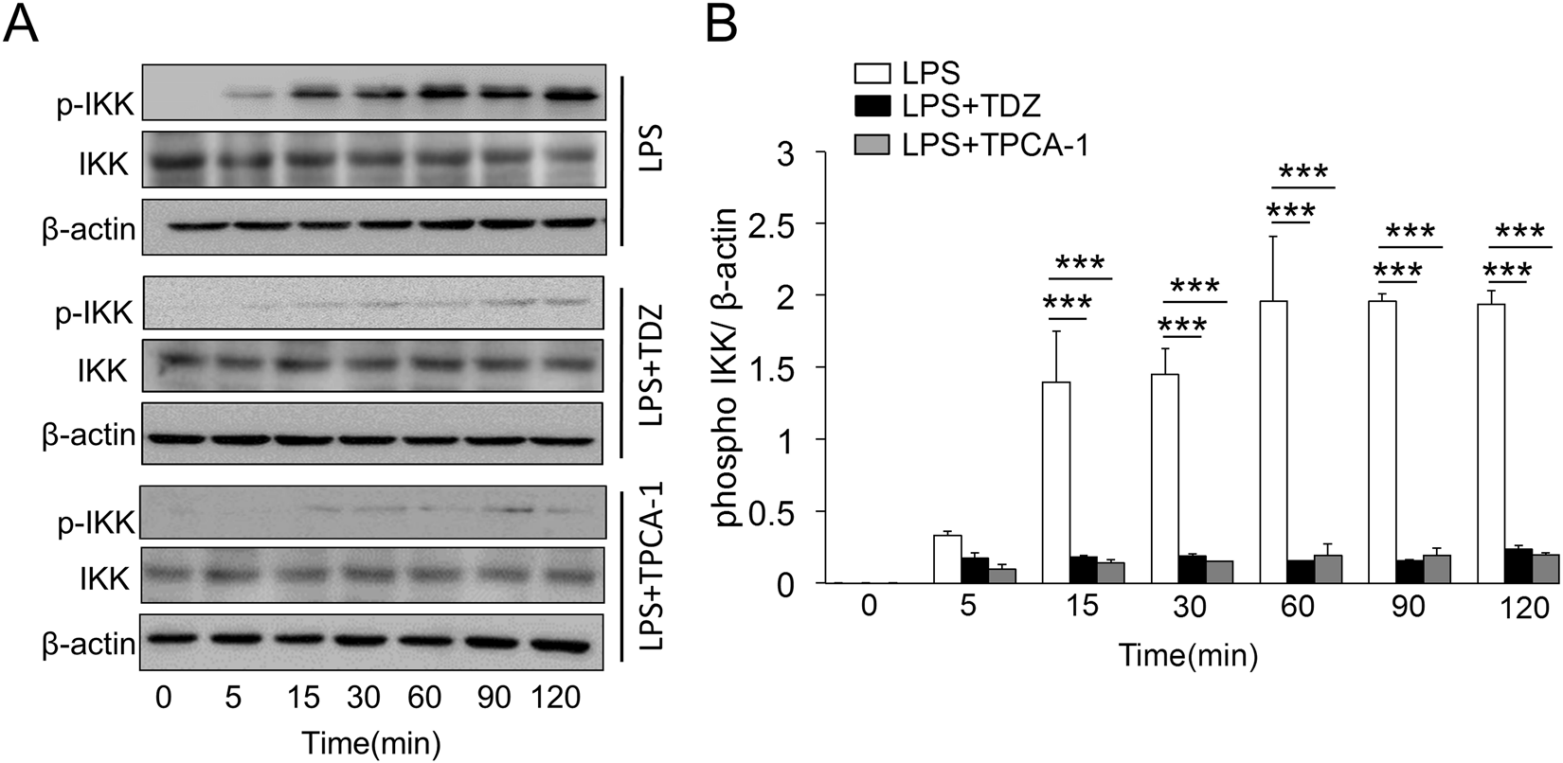
TPCA-1 inhibits phospho IKK and impedes the LPS mediated degradation of IkB. BMDM extracted from swiss albino mice were subjected to LPS (250 ng/ml) along with TDZ (10 μM) or TPCA-1 (1 μM). Post stimulation, proteins were extracted from lysed cells and subjected to immunoblot analysis **(A)** LPS stimulated BMDM immunoblotted for p-IKK, total-IKK and β-actin. **(B)** LPS along with TDZ stimulated BMDM immunoblotted for p-IKK, total-IKK and β-actin. **(C)** LPS along with TPCA-1 stimulated BMDM immunoblotted for p-IKK, total-IKK and β-actin. All data are representative of three independent experiments. ^***^P < 0.005.

To investigate the inhibitory effect of TDZ, RAW264.2 macrophages were treated with LPS in a time-dependent manner. LPS stimulation shows a significantly higher IKK phosphorylation at different time points. IKK activity increases just after 15 minutes of LPS stimulation on the other hand cells treated with TDZ show significant decrease in IKK activation (Fig. 7). The extent of IKK inhibition was determined using IKK known inhibitor TPCA-1. The inhibition studies show significant decrease of IKK inhibition as compared to TPCA-1.

### Inhibition of IKKβ Kinase activity by Thioridazine (TDZ)

**Figure 8:**
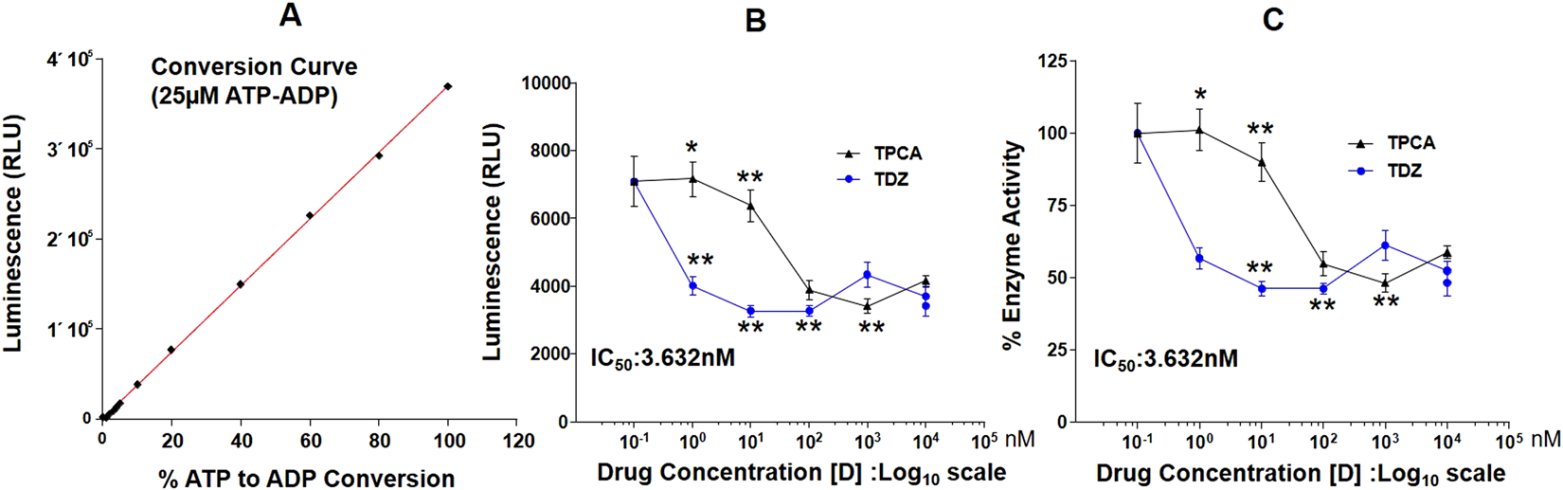
TDZ inhibits *in vitro* IKKβ Kinase activity. **(A)** Standard curve was prepared to determine the IKKβ activity **(B)** effect of TDZ on IKKβ Kinase inhibition was determined in comparison to the known IKKβ Kinase inhibitor TPCA-1. Data are expressed as the mean percentage of enzyme activity (or relative light units (RLU)) of the vehicle-treated control group (n = 7 wells). ^*^P < 0.005; ^**^<0.0005.

The inhibitory effect of TDZ on IKKβ Kinase was determined in comparison to the known IKKβ Kinase inhibitor, TPCA-1. Figure 8 A&B depicts the inhibition of IKKβ at various concentrations of TDZ and know IKKβ inhibitor TPCA-1. IC_50_ was determined for TDZ (3.632 nM), which is approximately 250 fold less than the IC_50_ determined for TPCA-1. The data clearly shows that TDZ is a very potent and selective inhibitor of IKKβ.

## Discussion

Inflammation is a physiological response of the body to tissue injury, pathogen invasion and irritants^26,27^. During the course of inflammation, immune cells of the innate and/or adaptive immune system are activated and recruited to the site of inflammation^28,29^. Attraction and activation of immune cells are regulated by a variety of cytokines and chemokines, which are predominantly regulated by transcription factors such as NF-κB, AP-1 and STATs^6,30,31^.

NF-κB is chronically activated in many inflammatory/immune diseases such as rheumatoid arthritis, cystic fibrosis and inflammatory bowel disease^32–34^. Therefore, the inhibition of NF-κB activation may be facilitated in a large number of human diseases, including cancer and many immune-mediated inflammatory diseases^35–37^. NF-κB activation relies on the phosphorylation of IκB proteins by IκB kinase (IKK). NF-κB is held in the cytoplasm in an inactive state by IκB inhibitors^38^. Inflammatory activation of NF-κB is achieved by stimulus-induced ubiquitination and subsequent proteasome-mediated degradation of IκBα. Once released from the inhibitor, NF-κB/p65 enters the nucleus to promote transcription of pro-inflammatory cytokines^5,39,40^. IκB kinase (IKK) is the convergent point in most signaling pathways activated by many stimuli leading to the inducible phosphorylation and degradation of IκB. Thus, a selective inhibitor of IKK would be of great interest as a potential anti-inflammatory agent. In the current study, we have used the approach of drug repurposing, where we used FDA approved drug data bank [http://www.epa.gov/nheerl/dsstox/] to discover a new role of existing drugs. We have identified a novel role of the anti-psychotic drug, TDZ, as an anti-inflammatory molecule. We further compared TDZ docking and binding properties with the known IKKβ inhibitor with high potencies, such as Bayer CA. Both compounds exquisitely superimposed with each other in the binding space of IKKβ (Fig. 1).

To validate our repurposing approach, we further tested the compound *in vivo* and determined the survival of mice with or without treatment of TDZ in LPS injected mice. Surprisingly, 80% of mice were survived in TDZ treated mice (Fig. 2). Interestingly, we found that lungs from TDZ treated mice were protected from LPS induced lung injury. This might be due to the IKK specific inhibition and IkB stabilization in the cytoplasm. Indirectly, we determined the IkB degradation by the total IkB protein content in the lung tissue as well as in Raw 264.7 macrophages using confocal microscopy (Figs 3 and 4). IkB stabilization leads to inhibition of NF-κB transcriptional activity by inhibiting its nuclear translocation^2,41,42^. Surprisingly, although IκB degradation was normal in macrophages treated with LPS, stabilization of the total protein level was observed after TDZ treatment. We further determined pro-inflammatory cytokine expression downstream of NF-κB, which clearly show the inhibition of NF-κB signaling in TDZ treated macrophages by stabilizing total IkB protein (Fig. 5). We also determined the proinflammatory cytokine expression in lung tissues of LPS treated mice in presence and absence of TDZ. We could see significantly downregulation of various cytokines in TDZ treated mice (Fig. 6). Further investigation confirmed that TDZ inhibits IKK phosphorylation and further downstream signaling of NFkB activation (Fig. 7). TDZ was found a potent inhibitor for IKKβ in *in vitro* kinase assay (Fig. 8). We have determined at 3.63 nM concentration, more than 50% inhibition could be achieved. In case of known IKKβ inhibitor (TPCA-1), same effect can be seen approximately at 900 nM concentration. This data strongly suggest that the TDZ is a potent specific inhibitor of IKKβ. IKKβ-dependent NF-κB activation is considered to play a major role in the transcriptional control of acute and chronic inflammation^40^, suggesting that IKKβ inhibitors may be effective anti-inflammatory drugs^43^. On the other hand, Greten *et al*.^44^, have also shown counter intuitively inhibition of this central pathway enhances susceptibility to endotoxin-induced shock and mortality by augmenting IL1β processing and secretion. These findings stand in sharp contrast to the marked ability of IKKβ inhibition to prevent TNFα expression and release, an endpoint that raised enthusiasm for targeting IKKβ in chronic inflammatory diseases such as rheumatoid arthritis and inflammation-induced bone loss^45^. Thus, in addition to revealing an unanticipated role for IKKβ-dependent NF-κB activation as a negative regulator of pro-IL1β processing, their results raise serious concerns about the long-term impact of IKKβ inhibition. Based on the current findings partial or initial inhibition of IKKβ inhibits IL1β expression on the other hand as per the Greten *et al*. study prolonged inhibition of IKKβ does seem to be as protective. The concept needs to be further evaluated to understand the molecular mechanisms in inflammatory response.

In summary, we have identified Thioridazineas a potent IKKβ inhibitor. It specifically inhibits IKK and stabilizes IkB, thereby inhibiting NF-κB mediated inflammatory signaling. This represents an important step towards the repurposing of this safe and widely available drug class for the treatment of inflammatory diseases.

## Electronic supplementary material

Supplementary Information
