# Supplementary material for: Repurposing Thioridazine (TDZ) as an anti-inflammatory agent
Source: Sci Rep. 2018 Aug 20;8:12471. doi: 10.1038/s41598-018-30763-5 (PMC6102213; doi:10.1038/s41598-018-30763-5)
Supplement: Supplementary file 1 — Supplementary Information [file 41598_2018_30763_MOESM1_ESM.pdf]

Repurposing Thioridazine (TDZ) as an anti-inflammatory agent

Mirza S. Baig[1, *], Anjali Roy[1], Uzma Saqib[2], Sajjan Rajput[1], Mansi Srivastava[1], Adnan Naim[1], Dongfang Liu[3], Rohit Saluja[4], Syed M. Faisal[5], Qiuwei Pan[6], Kati Turkowski[7], Gajanan N. Darwhekar[8], Rajkumar Savai[7,9, #]

[1]Discipline of Biosciences and Biomedical Engineering (BSBE), Indian Institute of Technology Indore (IITI), Simrol-453552, India.

[2]School of Basic Sciences, Discipline of Chemistry, Indian Institute of Technology Indore (IITI), Simrol-453552, India.

[3]Centre for Inflammation & Epigenetics, Houston Methodist Research Institute, Houston, Department of Microbiology and Immunology, Weill Cornell Medical College, Cornell University, New York, NY, USA.

[4]Department of Biochemistry, All India Institute of Medical Sciences (AIIMS),Bhopal-462 020, India.

[5]National Institute of Animal Biotechnology (NIAB), Hyderabad-500048, India

[6]Department of Gastroenterology and Hepatology, Erasmus MC-University Medical Center, 3015 CE Rotterdam, The Netherlands.

[7]Max Planck Institute for Heart and Lung Research, Department of Lung Development and Remodeling, 61231-Bad Nauheim, Germany.

[8]Acropolis Institute of Pharmaceutical Education and Research (AIPER), Indore-453771, India.

[9]Department of Internal Medicine, Universities of Giessen and Marburg Lung Center (UGMLC), member of the DZL, Justus Liebig University, Giessen 35392, Germany.

Running title: Repurposing of Thioridazine (TDZ)

*Corresponding Author: **Mirza S. Baig**,Discipline of Biosciences and Biomedical Engineering (BSBE), Indian Institute of Technology Indore (IITI), Indore- 453552, MP, India; Email: msb@iiti.ac.in;Tel: +91(7324) 306742.
#Co-corresponding Author:RajkumarSavai, Max Planck Institute for Heart and Lung Research, Department of Lung Development and Remodeling, 61231-Bad Nauheim, Germany; Email: rajkumar.savai@mpi-bn.mpg.de,Tel: +49 (6032) 705420.

Keywords: Repurposing,Inflammation, Macrophage, Nuclear factor-kB (NF-kB), IκB kinase (IKK), IκB, Thioridazine (TDZ)

Supplementary table 1. The top5 hits retrieved from the DockBlaster virtual screening program using the ZINC FDA approved database.

**Rank**	**ZINC ID**	**2-D Structure**	**Score kcal/mol**
1	1530695	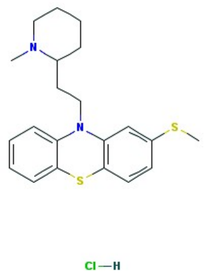	-52.49
2	3830847	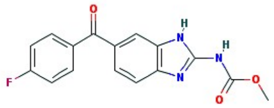	-52.27
3	599734	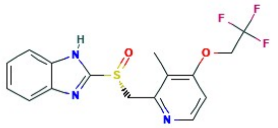	-51.47
4	57198	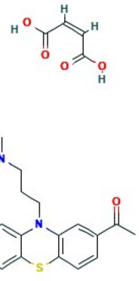	-49.66

5	538065	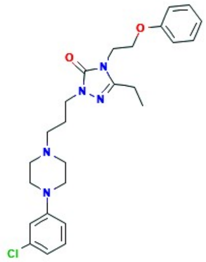	-49.62

Supplementary table 2. VinaDock scores of 8 known IKKβ inhibitors and top 5 FDA approved compounds.

PubChem ID	2-D Structure	General name	Vina Dock Score (mcule)	IKKβ inhibition (Ref.)
2807869	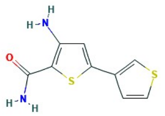	SC-514	-6.0	12
5081913	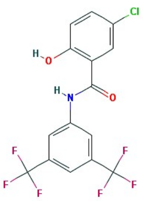	IMD-0354	-8.5	13
5281813	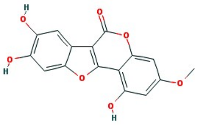	Wedelolactone	-8.6	14
9903786	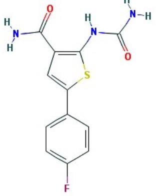	TPCA-1	-7.6	15
9813758	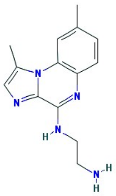	BMS-345541	-7.0	16

9929127	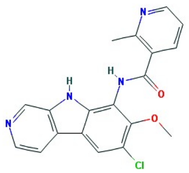	ML120B	-8.7	17
**20585253**	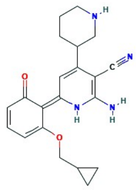	**Bayer Compound A**	**-8.4**	18
24983829	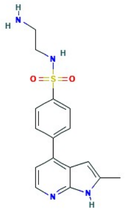	GSK-‘azaindole-7	-7.8	19
**ZINC 1530695**	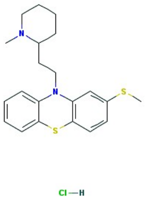	**Thioridazine Hydrochloride**	**-8.6**	-
ZINC 599734	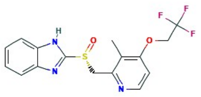	Dexlansoprazole	-7.7	-
ZINC 57198	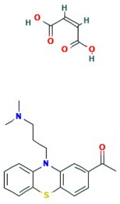	Acepromazine maleate	-7.6	-
ZINC 538065	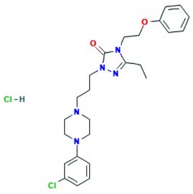	Nefazodone	-7.5	-

**ZINC 3830847**	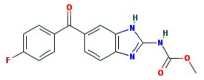	**Flubendazole**	**-8.8**	**-**

Supplementary Figure 1.

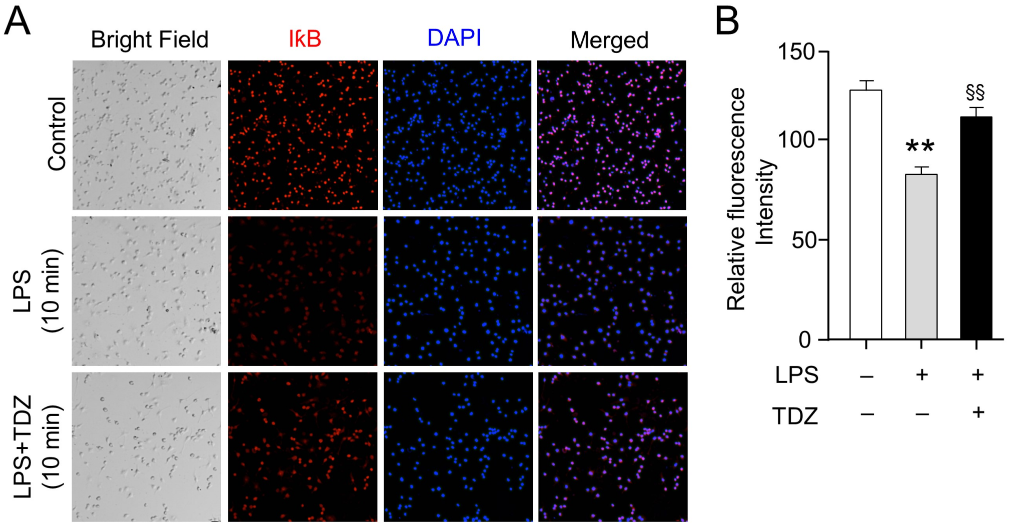

Supplementary Figure 1. TDZ inhibits LPS-induced IkB degradation in RAW 264.7 macrophages. (A) TDZ-induced restoration of IkB in the cell. LPS induce activation of downstream signalling pathway and phosphorylation of kinase protein like IKK. Once phosphorylated, IKK activates further phosphorylation of IkB followed by its degradation as seen in LPS (250ng/ml) treated cells in 10 mins. Prior treatment with TDZ which binds to activation loop within the kinase domain of IKK, inhibits IkB phosphorylation induced proteosome-mediated degradation at 10 min. Images are captured using Olympus microscopy at 10X magnification and 3 z zoom. (B) Intensity quantification of IkB. Each bar represents the intensity of 5 slices. Values are a mean $\pm$ standard error. *$P<0.05$.
